# Description of *Deladenus gilanica* n. sp. (Hexatylina: Neotylenchidae) isolated from wood of black pine in Northern Iran

**DOI:** 10.21307/jofnem-2020-065

**Published:** 2020-07-28

**Authors:** Parisa Jalalinasab, Mehrab Esmaeili, Weimin Ye, Ramin Heydari

**Affiliations:** 1 Department of Plant Protection, College of Agriculture and Natural Resources, University of Tehran, Karaj, Iran; 2 Nematode Assay Section, Agronomic Division, North Carolina Department of Agriculture and Consumer Services, Raleigh, NC 27607

**Keywords:** Molecular, Morphology, Morphometrics, Nematode, New species, rRNA gene

## Abstract

A new species of the genus *Deladenus*, *D. gilanica* n. sp. collected from Siahkal forests of Northern Iran, is described and illustrated. The new species is characterized by its 314 to 422-µm-long body, eight incisures in the lateral field, 7.5 to 8.0-μm-long stylet, position of the excretory pore, at the level of the hemizonid, both posterior to the nerve ring 61 to 76 µm from the anterior end, and conical tail with pointed tip. Based on morphology and morphometrics, the new species can be compared with eight known species of the genus: *D*. *aridus*, *D*. *durus*, *D*. *obesus*, *D*. *oryzae*, *D*. *processus*, *D. wilsoni*, *D. proximus*, and *D. posteroporus*. Evolutionary relationships of the new species with other *Deladenus* species were assessed with sequences of the D2 to D3 expansion regions of 28S rRNA and partial 18S rRNA gene. The phylogenetic analysis showed that *D*. *gilanica* n. sp. is genetically distinct from other included species. *Deladenus gilanica* n. sp. is a member of the *D. siricidicola* species complex and close to *D*. *canii*, *D*. *nitobei*, and *D*. *siricidicola*. Typologically, these species share the relative position of the excretory pore to the hemizonid.

The genus *Deladenus* (Thorne, 1941) belongs to the family Neotylenchidae (Thorne, 1941) with *D. durus* (Cobb, 1922; Thorne, 1941) as type species. It is characterized by the location of the esophageal-intestinal junction immediately behind the nerve ring, a median esophageal chamber sometimes present, and absence of a post-uterine sac. Two biological forms have been observed in this genus: the free-living form (mycetophagous stage) and the insect-parasitic form (infective stage). The mycetophagous stage has been observed in all the members of the genus. Bedding (1968) identified two species (*D. wilsoni* and *D. siricidicola*) with dimorphic females and proved a two-part life cycle, the free-living mycetophagous and the insect-parasitic phases. Later he identified other five entomophagous-mycetophagous species (Bedding, 1974). Blinova and Korenchenko (1986) proposed the genus *Beddingia* and family Phaenopsitylenchidae for the species with a known infective stage. Chitambar (1991) reviewed the mycetophagous stage of this genus, synonymized the genus *Beddingia* with *Deladenus*, and provided a diagrammatic classification in mycetophagous females of this genus based on the position of the excretory and hemizonid relative to each other (either posterior or anterior). According to this classification, all species except *D. norimbergensis* (Rühm, 1956), *D. aenea* (Rao and Reddy, 1982; Ebsary, 1991) and *D. ulani* (Sultanalieva, 1983) without information about the position of excretory pore relative to the hemizonid can be grouped into two categories: those with the hemizonid posterior to the excretory pore and species with it in anterior position. Currently, the genus *Deladenus* has 30 valid species. Three species have been reported from Iran: *D. durus* (Jahanshahi Afshar et al., 2014), *D. persicus* (Miraeiz et al., 2017), and *D. apopkaetus* (Chitambar, 1991) (Miraeiz et al., 2017).

During our surveys on plant-parasitic and free-living nematodes in Northern Iran, a new species of *Deladenus* was recovered from a wood sample of a dead pine tree (*Pinus niger* L.) in Siahkal region, Guilan Province, Northern Iran. The observations revealed that this population appeared to be morphologically and morphometrically distinct from any existing *Deladenus* species and it is herein described as *D. gilanica* n. sp.

The objectives of the present study were: (i) to provide an accurate description of the new species by an integrative approach to morphological and molecular characterization using the partial 18S and 28S D2 to D3 rRNA gene sequences, and (ii) to investigate the phylogenetic relationships of this neotylenchid nematode within the superfamily Sphaerularioidea.

## Materials and methods

### Sampling, extraction, mounting, and drawing

Soil, root, and wood samples were randomly collected from different regions of eastern forests in Guilan Province, Northern Iran during 2015. Nematodes were recovered directly from the wood samples by the Whitehead tray method (Whitehead and Hemming, 1965). The extracted nematodes were observed and handpicked under a stereomicroscope. Adult specimens for microscopic observation were killed with gentle heat and fixed in a solution of FGA 4:1:1 (formaldehyde, glycerin, and acetic acid) and then processed to anhydrous glycerin (De Grisse, 1969). Permanent slides were made and examined with a Nikon E200 light microscope. Morphometric data were obtained with the aid of a drawing tube attached to an Olympus BH2 light microscope. Photomicrographs were taken with a digital camera attached to an Olympus BH2 light microscope. Line drawings were made and photographs were taken with a digital camera attached to an Olympus BH2 microscope.

### DNA extraction, PCR, and sequencing

Single nematode specimens were handpicked and with light microscopy and then individually transferred to 10 μl distilled water on a glass microscope slide, crushed with a pipette tip and collected in 50 μl AE buffer (10 mM Tris-Cl, 0.5 mM EDTA; pH 9.0; Qiagen, Valencia, CA, USA) by pipette. DNA extracts were stored at −20°C until used as template for PCR amplification. The D2/D3 expansion segment of 28S rRNA gene was amplified using the forward D2A (5´–ACAAGTACCGTGAGGGAAAGTTG–3´) and reverse D3B (5´–TCGGAAGGAACCAGCTACTA–3´) primers (Nunn, 1992) and the partial 18S was amplified using primers 1096F (5´–GGTAATTCTGGAGCTAATAC-3´) and 1912R (5´–TTTACG GTCAGAACTAGGG–3´) (Holterman et al., 2006).

PCR cycle conditions for all rDNA regions were as follows: one cycle of 94°C for 2 min, followed by 35 cycles of 94°C for 30 s, annealing temperature of 55°C for 45 s, 72°C for 3 min, and finally one cycle of 72°C for 10 min. PCR products were purified after amplification using ExoSAP-IT (Affmetrix, USB Products), quantified using a Nanodrop spectrophotometer (Nanodrop Technologies) and used for direct sequencing in both directions using the primers referred to above.

### Phylogenetic analyses

The sequences were deposited into the GenBank database. DNA sequences were edited with ChromasPro1.5 2003-2009 (Technelysium Pty Ltd, Helensvale, Australia) and aligned using ClustalW (http://workbench.sdsc.edu; Bioinformatics and Computational Biology Group, Department of Bioengineering, UC San Diego, CA). All available species of *Deladenus* and some other Hexatylina species from GenBank were also selected for phylogenetic analysis. Outgroup taxa for each data set was chosen according to previous published data (Esmaeili et al., 2016). The model of base substitution in the sequences data were evaluated using MODELTEST version 3.06 (Posada and Criandall, 1998) based on the Akaike-supported model (Arnold, 2010). Bayesian analysis was performed to confirm the tree topology for each gene separately using MrBayes 3.1.0 (Huelsenbeck and Ronquist, 2001) running the chain for 1,000,000 generations and setting the ‘burnin’ at 1,000. Markov Chain Monte Carlo methods were used within a Bayesian framework to estimate the posterior probabilities (pp) of the phylogenetic trees (Larget and Simon, 1999) using the 50% majority rule. The *λ*^2^ test for homogeneity of base frequencies and phylogenetic trees was performed using PAUP* version 4.0 (Sinauer Associates, Inc., Publishers, Sunderland, MA).

## Results

### *Deladenus gilanica* n. sp.

**Figure 1:**
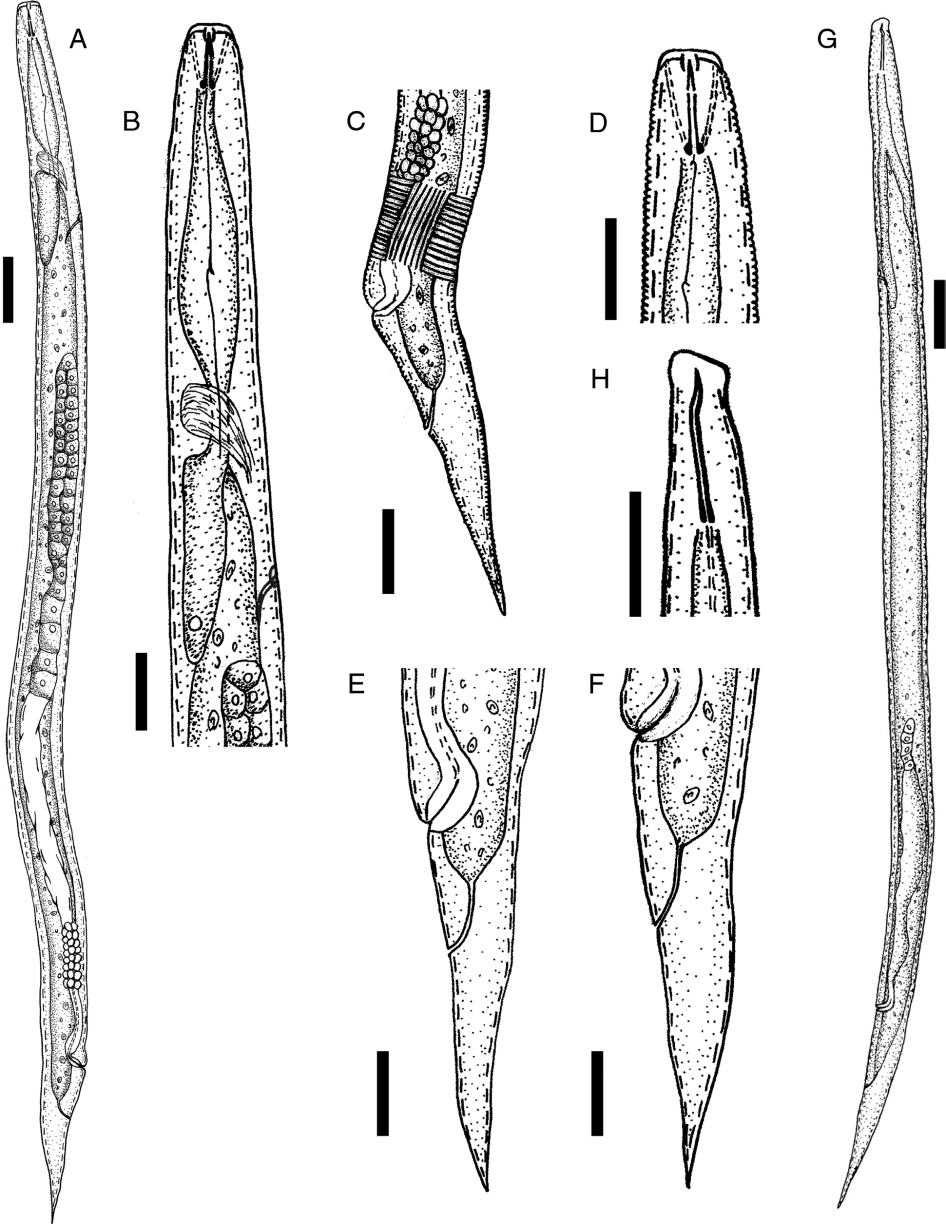
Line drawings of *Deladenus gilanica* n. sp. (A-F) Mycophagous female − (A) entire body; (B) pharyngeal region; (C) vulva region; (D) anterior end; (E, F) posterior body (tail). (G and H) Infective female − (G) entire body; (H) anterior end (Scale bars: A, G = 20 µm, B-F, H = 10 µm).

**Figure 2:**
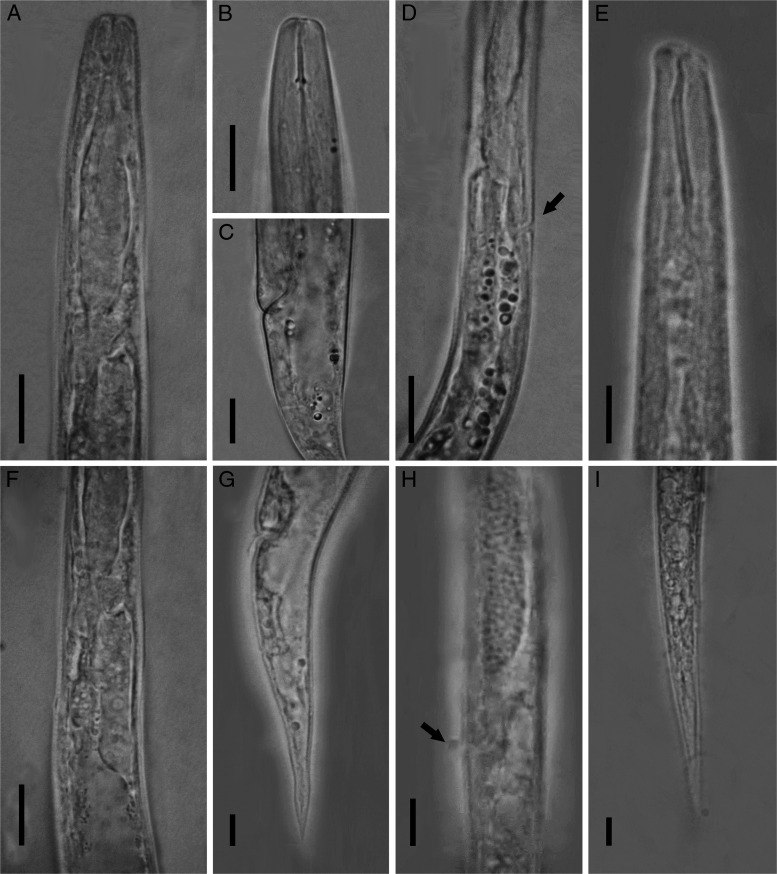
Photomicrographs of *Deladenus gilanica* n. sp. (A-D, F and G) Mycophagous female − (A) pharyngeal region; (B) anterior end; (C) vulva region; (D) pharyngeal region showing excretory pore and hemizonid; (F) end of female pharyngeal region; (G) female posterior body (tail). (E, H, and I) Infective female − (E) anterior end; (H) vulva region; (I) posterior body (tail) (All scale bars = 10 µm).

Figs. 1 and 2 show line drawings of *D. gilanica* n. sp.

### Measurements

Specific measurements are provided in Table 1.

**Table 1.** Morphometrics of female of *Deladenus gilanica* n. sp. from Iran.

	Mycetophagous stage		Infective stage
Characters	Holotype female	Paratype females	Paratype females
*n*	−	10	4
L	367	364.8 ± 33.7 (314-422)	330.3 ± 17.6 (310-341)
L'	361	335.4 ± 33.3 (285-393)	294.7 ± 15.3 (277-304)
a	18.6	21.1 ± 2.6 (18.1-26.5)	28.3 ± 0.1 (28.2-28.4)
b	8.5	7.1 ± 0.9 (5.9-8.5)	6.2 ± 1.8 (5.1-8.3)
b'	5.7	5.0 ± 0.9 (4.1-6.9)	3.7 ± 0.4 (3.4-4.1)
c	13.4	12.4 ± 1.1 (10.8-14.6)	9.3 ± 0.3 (9.0-9.4)
c'	2.4	3.3 ± 0.4 (2.4-3.8)	5.1 ± 0.4 (4.7-5.4)
V	87.4	86.6 ± 0.7 (85.4-87.4)	82.6 ± 0.6 (82.1-83.2)
Head height	1	1.4 ± 0.2 (1-1.5)	2.1 ± 0.3 (2-2.5)
Head width	6	6.5 ± 0.5 (6-7)	4.8 ± 0.3 (4.5-5)
Stylet	8	8.0 ± 0.2 (7.5-8.0)	13.9 ± 0.9 (13-15)
Excretory pore	71	68.4 ± 5.2 (61-76)	69 ± 6.5 (63-76)
Hemizonid	71	68.4 ± 5.2 (61-76)	68.5 ± 6.6 (63-76)
Pharynx	46	51.4 ± 4.9 (46-63)	54 ± 10.6 (41-64)
Overlapping	22	23.1 ± 7.5 (11-33)	32.3 ± 5.9 (29-41)
Body width	21	17.5 ± 2.8 (14-22)	11.8 ± 0.5 (11-12)
Head-vulva	341	316.0 ± 30.8 (268-367)	272.7 ± 12.7 (258-281)
Vulva-anus	20	19.4 ± 3.4 (15-26)	22 ± 3.0 (19-25)
Tail	29	29.4 ± 1.3 (27-32)	35.7 ± 2.5 (33-38)

**Note:** All measurements are in μm and in the form: Mean ± SD (range).

### Description

#### Mycophagous female

These are small nematodes with cylindrical body, gradually tapered toward both ends and straight or slightly curved upon fixation. Cuticle is with fine transverse striae, annulations less than 1 µm apart. Lateral field is with eight lines near vulva, reduced to four lines anteriorly and near the tail. Cephalic region is low, flattened, with rounded sides and continuous with body contour. Stylet is short, with distinct and posteriorly directed basal knobs. Conus occupies ca 33 to 47% of its total length. Orifice of dorsal pharyngeal gland is 0.5 to 1 μm posterior to stylet knobs. Pharynx is with a fusiform corpus, valveless, without distinct metacorpus. Subventral gland orifice is halfway between stylet basal knobs and the pharynx-intestine junction. Isthmus is narrow, short, and surrounded by nerve ring. Dorsal pharyngeal gland is short, overlapping intestine dorsally, with only one nucleus seen. Excretory pore is 71 μm (holotype) from anterior end. Hemizonid is at the level of excretory pore. Deirids is distinct, 1 to 13 μm posterior to hemizonid. Nerve ring and barely visible pharyngo-intestinal junction are either overlapping each other or junction is sometimes slightly posterior to nerve ring. Reproductive system is monodelphic and ovary is outstretched with two to three rows of oocytes in the multiplication zone. Spermatheca is invisible, crustaformeria is made up 8 to 12 columns of cells. Vagina is oblique, directed anteriorly. Vulva is a broad transverse slit, vulval lips nonprotuberant. Post-uterine sac is absent. Vulva-anus distance is less than tail length. Rectum and anus are distinct. Tail is conical, gradually tapering toward a pointed tip 2.4 to 3.8 times of the body width at the anus.

#### Male

Not found.

#### Infective female

These are small nematodes with almost straight body upon fixation. Cuticle is with fine transverse striae. Cephalic region is higher than mycophagous females, asymmetric, and continuous with body contour. Stylet is 13 to 15 μm long, with a wide lumen, without basal knobs but with a bit of inflation. The tip of the stylet is bent. Pharynx is with approximately cylindrical corpus and basal pharyngeal bulb developed with dorsal and ventral gland nuclei. Excretory pore is 63 to 76 μm from anterior end. Hemizonid is at the level of excretory pore or just anterior to that. Reproductive system is monodelphic and ovary is short with short germinal zone. Vagina is oblique, directed anteriorly, vulval lips nonprotuberant. Post-uterine sac is absent. Vulva-anus distance is less than tail length. Tail is conical, gradually tapering toward a pointed tip 4.7 to 5.4 times of the body width at the anus.

### Type host and locality

*D. gilanica* n. sp. was obtained from the wood samples of a dead black pine tree (*Pinus nigra*) of Siahkal region in Guilan Province, Northern Iran (GPS coordinates: N 37° 64´, E 49° 51´).

### Type material

Holotype female, three paratype mycetophagus females, and two infective females (Slides NDG001 and NDGR002, NDG006) deposited at Nematode Collection of Department of Plant Protection, College of Agricultural and Natural Resources, University of Tehran, Karaj, Iran. Four female paratypes deposited at National Nematode Collection of the Department of Nematology, Iranian Research Institute of Plant protection, Tehran, Iran. Two paratype mycetophagus females and two infective females deposited in USDA Nematode Collection, Beltsville, MD, USA.

### Etymology

The specific epithet refers to the province of occurrence of the new species.

### Diagnosis and relationships

The new species (based on the mycetophagous stage) is characterized by body length of 314 to 422 μm long, eight incisures in the lateral field and the position of the excretory pore and hemizonid at the same level (see Table 2).

**Table 2.** Comparison of *Deladenus gilanica* n. sp. with eight similar species.

Species	L	a	b	c	V	Stylet	Median corporeal chamber	Excretory pore relative to hemizonid^a^	Tail	Li^b^	Male	Infective stage
*D. gilanica* n. sp.	314-422	18.1-26.5	5.9-8.5	10.8-14.6	85.4-87.4	7.5-8	–	SL	27-32	8 at level of vulva	–	+
*D*. *durus*	620-13,60	21-50	7.7-16	19.5-39.3	90-95	6-11	+	P	21-43	6-7	+	–
*D*. *aridus*	693	31.2		19.8	91.9	9.4	–	P		5	–	–
*D*. *obesus*	970-1,400	18-26	8-16.9	32-33.6	92-93	7.3-9	–	P	30-47	8-10	–	–
*D*. *processus*	760-990	34-49	13.3-17.7	19.6-22.8	92.2-93.5	6-7	–	P	35-44	6	+	–
*D*. *oryzae*	660-960	19-26	7.9	29	92-93.5	7-9	+	A	29-34	Indistinct	+	+
*D. wilsoni*	1,490-2,700	34.6-62.6	15.5-26.5	46.6-66.9	94.6-96.2	10-11	–	A or SL		7-9 at level of vulva	+	+
*D. proximus*	1,760-2,200	40-53.7	16.3-21.3	44-60	95.1-95.9	11-12	–	A	31-47	7-8 at level of vulva	+	+
*D. posteroporus*	889-1,026	38-43	10.1-12.8	31.3-37.1	93.5-95.9	8-11	–	A	27-29	11-12 at midbody	+	+

**Notes:** Measurements are in μm and in the form: range and/or (mean). ^a^A, anterior, P, posterior, SL, at the same level; ^b^number of lateral incisures.

*Deladenus gilanica* n. sp. differs from all other described *Deladenus* species by the shorter body and the position of the excretory pore relative to the hemizonid. Due to the position of excretory pore and hemizonid, the new species can be compared with some species like *D*. *durus*, *D*. *aridus* (Andrássy, 1957), *D*. *obesus* (Thorne, 1941), *D*. *processus* (Tomar et al., 2015), *D*. *oryzae* (Bajaj, 2015), *D. wilsoni*, *D. proximus* (Bedding, 1974), and *D*. *posteroporus* (Yu *et al*., 2017). It can be distinguished from these species by having a shorter body, position of the excretory pore and hemizonid, anteriorly located vulva (less V index), longer and conical tail (less c index) with pointed tip and shape of lip region, and also by the stylet and tail shape in infective females.

### Molecular phylogeny and discussion

**Figure 3:**
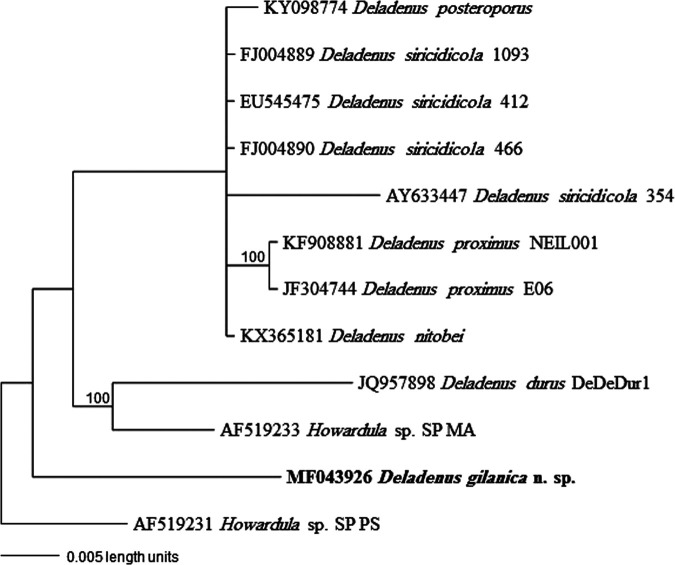
The 10001st Bayesian tree inferred from 18S under GTR + I + G model (−lnL = 3768.1687; AIC = 7556.3374; freqA = 0.2621; freqC = 0.1952; freqG = 0.2673; freqT = 0.2755; R(a) = 1.4504; R(b) = 3.0092; R(c) = 1.5594; R(d) = 0.3743; R(e) = 7.8701; R(f) = 1; Pinva = 0.4966; Shape = 0.6306). Posterior probability values exceeding 50% are given on appropriate clades.

**Figure 4:**
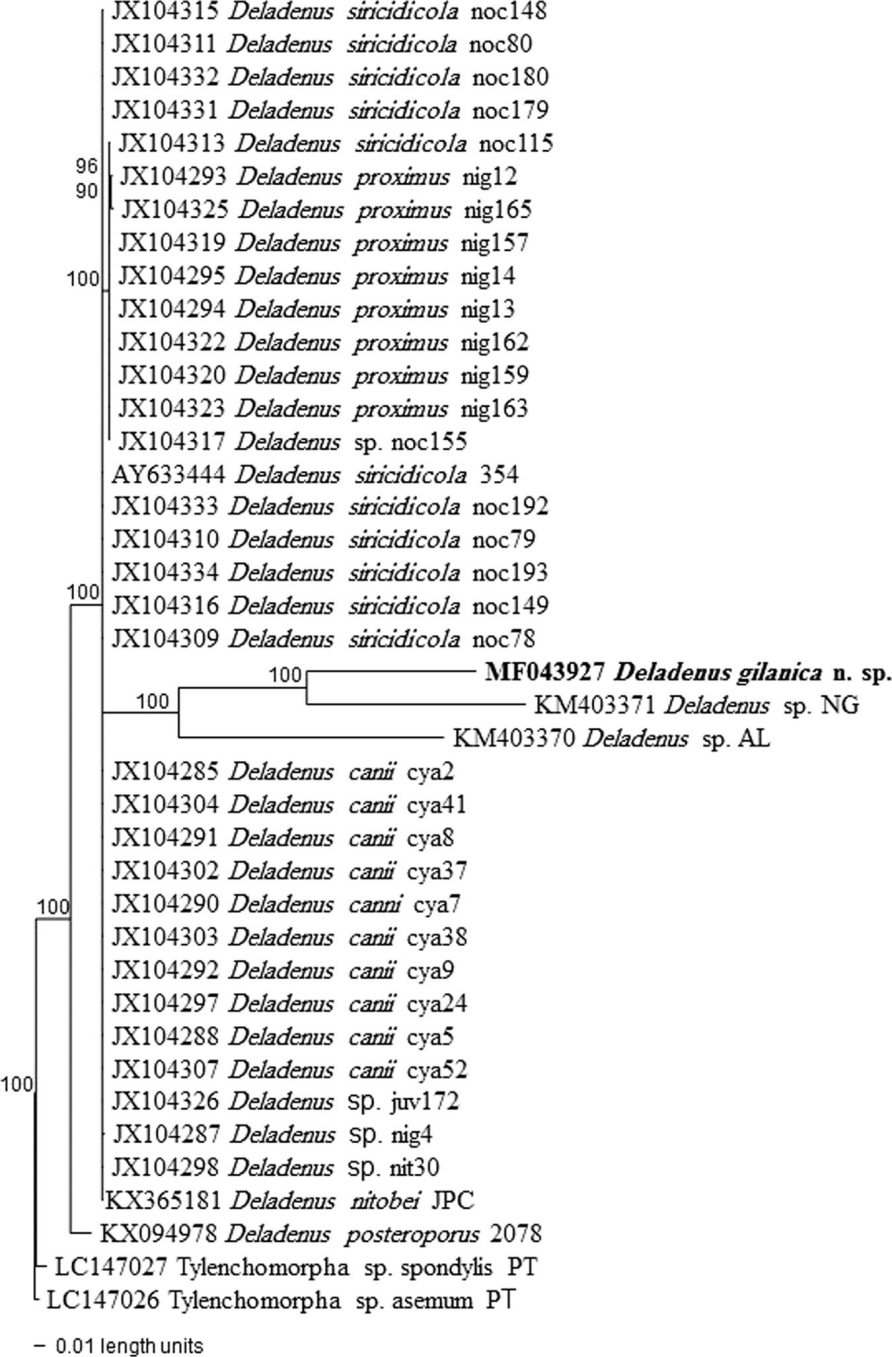
The 10001st Bayesian tree inferred from Revised 28S D2/D3 under TVM + I + G model (−lnL = 3258.8145; AIC = 6535.6289; freqA = 0.1843; freqC = 0.2082; freqG = 0.3284; freqT = 0.2791; R(a) = 1.3629; R(b) = 4.8955; R(c) = 2.5959; R(d) = 0.7212; R(e) = 4.8955; R(f) = 1; Pinva = 0.2327; Shape = 0.4722). Posterior probability values exceeding 50% are given on appropriate clades.

Amplification of the partial 18S and 28S D2/D3 rRNA gene sequences from *D. gilanica* n. sp. specimens yielded single fragments of approximately 900 and 800 bp, respectively, based on gel size. The partial 18S rRNA gene sequence of *D. gilanica* n. sp. (GenBank Accession No. MF043926) was less than 96% homologous to any available DNA sequences from GenBank. The BlastN search revealed the highest match with sequences of a member of the Neotylenchidae (KY907662) and several isolates of *Fergusobia leptospermum* (Davies et al., 2018) (KX611458, FJ393268, AY589292, FJ393270, AY589303, KF209340) with 95 to 96% identity, with substitutions ranging from 50 to 65 and from 6 to 13 indels. The 28S D2/D3 sequence of *D. gilanica* n. sp. (MF043927) was less than 88% homologous with any available DNA sequences from GenBank. The BlastN search revealed the highest match with *D*. *posteroporus* (KX094978) and an undescribed/unidentified species of the genus (JX104313) with 44 to 60 substitutions and 8 to 15 indels (Figs. 3 and 4).

Molecular phylogenetic analysis based on rRNA gene sequences suggested that the new species belongs to the *Deladenus* clade with 100% bootstrap support, but the status within the genus was not clearly resolved. Although posterior probability support was not high for any *Deladenus* clade besides that for the three isolates of *D. siricidicola*, the inferred tree largely agrees with the tree of Kanzaki et al. (2016, 2017).

Phylograms reveal that the new species is close to *D*. *siricidicola* complex/clade (*D*. *canii*, Bedding, 1968; *D*. *nitobei*, Kanzaki et al., 2016, and *D*. *siricidicola*); all share the position of the excretory pore relative to the hemizonid and support the morphological characteristics. The vast majority of described *Deladenus* species have not yet been sequenced, and thus inference phylogenetic relationships will require molecular characterization of further species.

Among the nominal species, the position of the excretory pore differs dramatically, either relative to the anterior end, or to the hemizonid. Its position relative to the hemizonid has been documented as the most important diagnostic character to differentiate species (Chitambar, 1991; Yu et al., 2013). All the putative dimorphic species have the excretory pore anterior to the hemizonid, while almost all of the other presumed non-dimorphic species, with the exception of *D*. *apopkaetus* (Chitambar, 1991), which have the excretory pore posterior to the hemizonid. The distances between the two structures vary between species of *Deladenus* and may overlap. We observed that the hemizonid and the excretory pore are located at the same level in *D. gilanica* n. sp., which is a unique character among reported species of the genus.

In addition to the morphological character (distance between the hemizonid and the excretory pore in the mycophagous form), Kanzaki et al. (2016) distinguished *D. nitobei* and *D. siricidicola* from the other *Deladenus* species reported from Japan by their preferences in host wasp and food source. Bedding and Akhurst (1978) reported that *Deladenus* species were fungus specific. A detailed field survey of the insect host-nematode-fungi associations is necessary to understand the bionomics of the new species.
